# Essential Oils: Chemistry and Food Applications

**DOI:** 10.3390/foods13071074

**Published:** 2024-04-01

**Authors:** Jesus M. Rodilla, Tiago Rosado, Eugenia Gallardo

**Affiliations:** 1 FibEnTech-UBI, Department of Chemistry, Universidade da Beira Interior, Rua Marques de Ávila e Bolama, 6201-001 Covilhã, Portugal; 2 Centro de Investigação em Ciências da Saúde (CICS-UBI), Universidade da Beira Interior, 6201-506 Covilhã, Portugal; 3 Laboratório de Farmaco-Toxicologia, UBIMedical, Universidade da Beira Interior, 6201-506 Covilhã, Portugal

In recent years, the crucial role played by essential oils in various areas such as health, cosmetics, crop protection, and food industries has been increasingly recognized. In terms of food applications, industries have been utilizing extracts of aromatic plants and essential oils due to their ability to control the growth of pathogenic microorganisms. It is essential to understand the mechanisms behind these effects (antimicrobial, antioxidant, and antifungal) and their interactions. The diverse properties of essential oils present the opportunity to use natural, safe, eco-friendly, cost-effective, renewable, and easily biodegradable antimicrobials for food preservation in the near future [1,2]. In this context, this Special Issue aims to address the latest advancements in scientific research on essential oils, including their applications as value-added compounds in the food industry. Subsequent to the peer review process, five original research articles were included in this Special Issue of *Foods*.

Cerrón-Mercado et al. (contribution 1) investigated the chemical composition, antioxidant, and antibacterial properties of essential oil extracted from *Tagetes elliptica* Sm. leaves, grown in Peru. Extracted via steam distillation, the oil’s composition was analyzed using GC-MS. Different antioxidant assays (DPPH, ABTS, FRAP, and FIC) and the Rancimat test were employed alongside the agar well diffusion method for antibacterial activity against *Staphylococcus aureus*, *Escherichia coli*, and *Salmonella infantis*. Twenty-seven compounds were identified, with cis-tagetenone, trans-tagetenone, dihydrotagetone, and trans-tagetone as major components. The essential oil displayed significant antioxidant activity, surpassing standard antioxidants’ efficacy. It exhibited potent antibacterial properties against all tested strains. This study underscores *T. elliptica* essential oil’s potential as a natural alternative to synthetic antioxidants and antimicrobial agents in the food industry. While previous research has noted the benefits of *Tagetes* essential oils, scant attention has been given to *T. elliptica*. Thus, this study fills a gap by elucidating its chemical composition and bioactive potential. Despite the need for further investigation into specific active compounds and their mechanisms, *T. elliptica* essential oil shows promise as a safe, eco-friendly food preservative. Further research is warranted to assess its efficacy in complex food matrices.

Encapsulation of essential oils faces challenges in material selection, retention of biological activity, long-term stability, and scalability. Emerging technologies like nanotechnology enhance efficiency and precision, enabling controlled release systems. Novel natural and biodegradable polymers expand applications. Despite progress, optimizing encapsulation conditions and ensuring stability remain key challenges. Continued research promises innovative solutions to maximize essential oils’ therapeutic potential. Indeed, encapsulation is the focus area of the study conducted by Kong et al. (contribution 2) and Prasad et al. (contribution 3). The study of Kong et al. (contribution 2) aimed to develop anti-*Staphylococcus aureus* inclusion complexes (ICs) using the Hinoki essential oil (HEO) complexed with β-cyclodextrin (β-CD) and 2-hydroxypropyl-β-cyclodextrin (2-HP-β-CD) through an ultrasound-assisted kneading method. Key findings include the successful preparation of HEO/β-CD and HEO/2-HP-β-CD inclusion complexes with high recovery yield, embedding fraction, and loading capacity. The ultrasound-assisted kneading method exhibited superior efficiency over the traditional method in complexation. Characterization of inclusion complexes using SEM, XRD, GC-MS, and FT-IR confirmed successful formation. Significant anti-*Staphylococcus aureus* activity of the inclusion complexes was demonstrated, with inhibitory rates reaching 99.8% and 100% at a dose of 20 g L^−1^ in a liquid culture medium. The hydrophilic nature of the inclusion complexes indicated the potential for blending with hydrophilic biodegradable materials for diverse food packaging applications. This study represents a novel approach to the development of green anti-*S. aureus* inclusion complexes using HEO, β-CD, and 2-HP-β-CD, with the ultrasound-assisted kneading method showing promise for efficient complexation. Further research is suggested to explore the release characteristics of HEO molecules from inclusion complexes under different environmental conditions. Prasad et al. (contribution 3) focused their study on encapsulating *Cymbopogon khasiana* × *Cymbopogon pendulus* essential oil (CKP-25-EO) into a chitosan nanoemulsion and evaluating its efficacy in inhibiting fungal growth and aflatoxin B1 (AFB1) contamination in *Syzygium cumini* seeds. Various analyses, including DLS, AFM, SEM, FTIR, and XRD, confirmed the successful encapsulation of CKP-25-EO in chitosan with controlled delivery. The encapsulated CKP-25-Ne exhibited enhanced antifungal, antiaflatoxigenic, and antioxidant activities compared to free essential oil. Cellular and molecular mechanisms of action were elucidated, showing inhibition of fungal ergosterol and methylglyoxal biosynthesis. In situ efficacy was demonstrated for inhibiting lipid peroxidation and AFB1 secretion in stored *S. cumini* seeds without altering sensory properties. The study concluded that CKP-25-Ne is a safe and effective green nano-preservative against fungal and AFB1 contamination in food, agriculture, and pharmaceutical industries. Encapsulation of CKP-25-EO in chitosan nanomatrix enhanced its antifungal and AFB1 mitigation abilities and its antioxidant properties. In silico modeling supported the mechanism of action by showing interaction with proteins involved in AFB1 biosynthesis. Overall, CKP-25-Ne showed promising potential as a natural preservative with practical applications in various industries.

A different approach was taken by Santos et al. (contribution 4). These authors explored biodegradable alternatives for packaging materials, thus minimizing the use of plastics. This study focused on addressing the global issue of plastic pollution by exploring biodegradable alternatives for packaging materials. The aim of this work was to produce and analyze k-carrageenan films infused with *Cymbopogon winterianus* essential oil, which exhibited significant antioxidant and antibacterial properties. The essential oil, primarily composed of citronellal, demonstrated remarkable antioxidant activity and inhibited the growth of *Listeria monocytogenes* bacteria. Scanning electron microscopy revealed reductions in *L. monocytogenes* biofilms when in contact with the developed films. Additionally, the essential oil showed potential in inhibiting quorum sensing, further enhancing its antibacterial effects. The transparent and slightly hydrophobic nature of the produced films and their bioactive properties make them promising candidates for eco-friendly packaging materials. The main innovation lies in the utilization of *C. winterianus* essential oil as a potential food preservative in k-carrageenan films, offering a sustainable alternative to conventional plastics. Future research should focus on increasing production and evaluating biodegradability under various conditions.

Another interesting study is the one developed by De Bruno et al. (contribution 5). This study aimed to assess the impact of applying edible coatings on the shelf life of strawberries, with a focus on extending availability and maintaining quality. Specifically, the study evaluated coatings enriched with natural antioxidants for their physicochemical, microbial, and structural properties during refrigerated storage for up to 14 days. Different concentrations of natural antioxidant extracts from bergamot pomace, bergamot essential oil, and a synthetic antioxidant (BHT) were incorporated into gum Arabic coatings alongside untreated strawberries as a control. The enriched coatings effectively preserved the strawberries’ qualitative parameters, with the 2.5% antioxidant extract and 0.1% bergamot essential oil demonstrating the best maintenance after 14 days. These samples exhibited lower decay rates, good consumer acceptability, and retained high levels of ascorbic acid (>30 mg to 100 g^−1^). The novelty of this research lies in formulating edible coatings with natural antioxidants derived from bergamot by-products, aligning with principles of circular economy and sustainability. The coatings effectively delayed ripening and senescence, providing a barrier against deterioration while preserving antioxidant parameters, thus enhancing the strawberries’ shelf life. The study underscores the potential of natural antioxidants and essential oils in edible coatings for extending fruit quality and safety during storage.

The research papers published in this Special Issue represent some of the innovative strategies at our disposal for characterizing and valorizing essential oils in the food industry. However, more studies are still needed to overcome some of the challenges essential oils pose, such as stability and the maintenance of their physicochemical and organoleptic characteristics. Additionally, more research and development are required to explore new applications, formulations, and processing technologies that optimize the efficacy and application of essential oils in the food industry.
